# AgeFactDB—the JenAge Ageing Factor Database—towards data integration in ageing research

**DOI:** 10.1093/nar/gkt1073

**Published:** 2013-11-11

**Authors:** Rolf Hühne, Torsten Thalheim, Jürgen Sühnel

**Affiliations:** Biocomputing Group, Leibniz Institute for Age Research – Fritz Lipmann Institute, Jena Centre for Systems Biology of Ageing – JenAge, Beutenbergstrasse 11, Jena, Germany

## Abstract

AgeFactDB (http://agefactdb.jenage.de) is a database aimed at the collection and integration of ageing phenotype data including lifespan information. Ageing factors are considered to be genes, chemical compounds or other factors such as dietary restriction, whose action results in a changed lifespan or another ageing phenotype. Any information related to the effects of ageing factors is called an observation and is presented on observation pages. To provide concise access to the complete information for a particular ageing factor, corresponding observations are also summarized on ageing factor pages. In a first step, ageing-related data were primarily taken from existing databases such as the Ageing Gene Database—GenAge, the Lifespan Observations Database and the Dietary Restriction Gene Database—GenDR. In addition, we have started to include new ageing-related information. Based on homology data taken from the HomoloGene Database, AgeFactDB also provides observation and ageing factor pages of genes that are homologous to known ageing-related genes. These homologues are considered as candidate or putative ageing-related genes. AgeFactDB offers a variety of search and browse options, and also allows the download of ageing factor or observation lists in TSV, CSV and XML formats.

## INTRODUCTION

The continuing increases in life expectancy pose a demanding challenge to society in general and public health systems in particular. Promotion of health in old age requires intensive research efforts into both the processes of ageing and age-related diseases. Ageing is an extremely complex phenomenon. With conflicting hypotheses on the molecular and physiological basis (1) and even the origin (2), a unified molecular model which explains ageing is currently missing from the field. A reductionist approach is inadequate to understand the functional changes associated with ageing and therefore, strategies suited to study complex systems are required (3). System-wide studies, using integrative multi-disciplinary projects and the correct integration of already known single-piece information could help towards this aim. We believe that research on ageing and associated diseases will benefit greatly from such a systems biology or integrative biological approach.

One important aspect of this development would be integration of data taken either from existing databases or directly from the scientific literature. The establishment of databases was one of the consequences of the data explosion seen especially in molecular biology. Which, although not initially popular are necessary tools for life science research. Recently, in addition to the very useful general databases, further ageing-specific databases have been launched (a fairly comprehensive compilation of such resources can be found in the database section of the JenAge Information Centre http://info-centre.jenage.de). The JenAge information hub assists researchers working on ageing and age-related diseases as well as in systems biology, and includes in the database section the four categories: biological data, demographic data, diseases and metadata. The biological data category currently has 25 entries. Among them there are, however, five databases that seem to be no longer active.

Pioneering work in the ageing databases field was done by Joao Pedro de Magalhaes with the development of the Human Ageing Genomics Resources (4,5). We have a long-standing expertise in the development of structural biology (6) and genomics (7–10) resources and this will, in our opinion, be fruitful for databases on the biology of ageing. Therefore, we have developed a new database, AgeFactDB, that integrates information on ageing factors (genes, chemical compounds and environmental or lifestyle cues, e.g. calorie restriction).

The main focus of the new database is data integration. With previous attempts at data integration for biological ageing data having either been discontinued (11) or being at the level of simple crosslinking between different resources (4), AgeFactDB is a more advanced database with regard to data integration that combines data as far as possible with a single interface.

## BASIC CONCEPT AND TERMINOLOGY

The JenAge Ageing Factor Database (AgeFactDB) is aimed at the collection and integration of ageing-related data. Currently, we include primarily data on ‘Ageing Factors’. These factors are genes, chemical compounds or other environmental cues such as calorie restriction, for example, whose effect is a lifespan change or another ageing phenotype under specific conditions. Information related to the effects of ageing factors is called an ‘Observation’ and represents ageing-related evidence. Note, that in our definition the determination of an ageing factor always includes the comparison of two different experimental setups. For example, the comparison of wild-type strains with gain-of-function or loss-of-function strains. In relation to the administration of chemical compounds not only the comparison between experiments with and without a chemical compound but also variations of concentrations are included. Finally, environmental factors such as dietary restriction or overfeeding also require comparisons to a normal diet.

However, when looking at the Lifespan Observation Database, one encounters a further type of data. In the case of the AgeFactDB observation OB_005029, which corresponds to observation 2775 in the Lifespan Observations Database, only the mean lifespan for male worms of strain N2 at 20°C is given as 18.1 days, with no comparison to another experimental setup is made (12). Meaning, that for this observation no ageing factor can be assigned, but because these data are obviously of relevance for ageing, AgeFactDB includes a number of observations of this type.

The basic idea of data integration is a 2-fold one. On one hand, AgeFactDB data integration aims to define, as far as possible, a uniform data structure for all data, irrespective of their sources. On the other hand, we want to provide as much source transparency as possible. Thus, source information for all inputs is on the database interface.

To identify ageing factors and observations in different releases, we provide a unique AgeFactDB ID (AF_nnnnnn) for each ageing factor and each observation (OB_nnnnnn), allowing the identification of ageing factors in cases where, for example, the name has changed in different releases.

## DATABASE CONTENT

**Figure 1.**
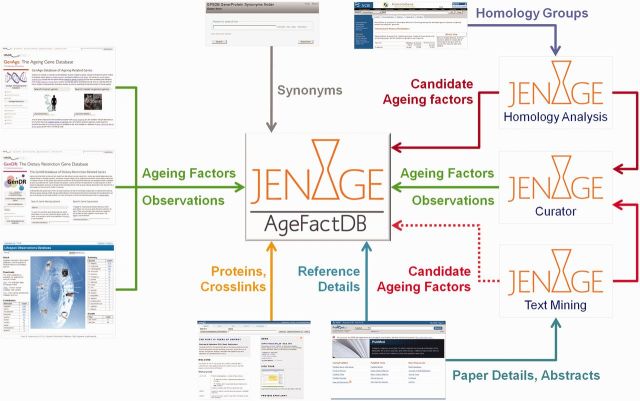
Data integration chart of AgeFactDB. The dotted line indicates future plans.

In a first step ageing-related data were primarily taken from existing databases, the Lifespan Observations Database (http://lifespan.sageweb.org), the Ageing Gene Database**—**GenAge and the Dietary Restriction Gene Database**—**GenDR (4,5). In addition, we have started to include new ageing-related information using both manual and semi-automatic information extraction from the scientific literature. It is important to note here that work on an automated text-mining pipeline in collaboration with the Jena University Language & Information Engineering Lab (JULIE Lab) is ongoing. Finally, for all experimentally known ageing-related genes homologues from the HomoloGene Database are included (13). They can be considered as candidates or putative ageing genes, but their ageing relevance evidence is of course only computational. Thus, AgeFactDB currently contains information from five ageing-related sources, the three databases mentioned earlier, the AgeFactDB Curator and from AgeFactDB Homology Analysis. Figure 1 illustrates the data flow in AgeFactDB. The dotted line indicates future plans. As of 15 August 2013 AgeFactDB includes experimental information on more than 2700 ageing factors based on data from more than 8000 observations. A more detailed content statistics of AgeFactDB ageing factors and observations can be found in Tables 1 and 2.

**Table 1.** AgeFactDB summary statistics of ageing factors as of 15 August 2013

Ageing factor type	Ageing relevance evidence			
	Experimental	Computational	Both	Any
Gene	2594	14 437	581	16 450
Compound	91	–	–	91
Other ageing factor	58	–	–	58
Total	2743	14 437	581	16 599

Ageing factors can have both experimental and computational ageing relevance evidence because they may include a number of different observations. These examples are included both in the experimental and computational categories. Their total sum is thus given by Experimental + Computational–Both.

**Table 2.** AgeFactDB summary statistics of ageing-related observations as of 15 August 2013

Type	Experimental	Computational	Any
Ageing phenotype—data type 1	940	–	940
Ageing phenotype—data type 2	7219	–	7219
Homology analysis	–	1452	1452
Total	8159	1452	9611

Contrary to ageing factors ageing-related observations are always either computational or experimental. Description of data types 1 and 2 is given in the ‘Data integration and validation’ section.

AgeFactDB also includes a number of observations in which genetic manipulations, the administration of chemical compounds or application of other factors did not result in ageing-related effects. Hence, these observations do not indicate an ageing relevance. For example, Shen *et al.* reported that the *ttx-3*, *sra-11*, *ceh-10* and *ceh-23* genes are required for the proper function of AIY interneurons (14). In further analysis, mutations in *ttx-3* and *ceh-10* significantly decreased lifespan, whereas mutations in *sra-11* and *ceh-23* did not affect lifespan. As no other experimental results for the *ceh-23* and *sra-11* genes are currently included in the AgeFactDB source databases, neither gene should be considered an ageing factor. However, in our opinion, their inclusion in the AgeFactDB will help to provide a better understanding of the ageing. Subsequently, upon closer inspection of the literature a more recent report on the *ceh-23* gene was found, showing that it mediates prolonged longevity in response to an impaired mitochondrial electron transport chain (15). The status of the *ceh-23* ageing factor will change following integration of these experimental results into the AgeFactDB.

It is a fairly typical situation that for ageing factors, there are observations that both support or refute a relevance to ageing, which may be a consequence of different experimental conditions. For example, the *Caenorhabditis elegans* gene *age-1* currently has nine observations where changes in function decreased lifespan, 65 studies with an increased lifespan and 9 with no lifespan effect. Finally, as mentioned earlier, AgeFactDB contains a few lifespan data taken from the Lifespan Observations Database that do not refer to the intervention of an ageing factor.

## DATA INTEGRATION AND VALIDATION

Data integration is usually hampered by the fact that the data structures of the sources to be integrated are different, a problem often encountered here. The GenDR and GenAge databases provide the major part of information as free text, whereas the Lifespan Observation Database offers, in addition to a free-text description, separate fields for temperature, lifespan, reference lifespan and lifespan change. In the most recent version of GenAge there are also some additional lifespan-related data fields, which differ from the data structure of the Lifespan Observations Database. This means that to produce a unified data structure in AgeFactDB parsing of the free text descriptions and extraction and expansion of quantitative data are required, needing a large manual curation effort that we have postponed to a later stage. Therefore, AgeFactDB offers ageing phenotype information in two formats (Type 1 and Type 2). Observations either contain the data mostly un-separated within a single description (Type 1) or contain lifespan data in a more structured form with separate fields (e.g. lifespan effect, lifespan change and lifespan value**—**Type 2). However, we have already taken a step towards data structure unification by separating automatically data combined within GenAge in a single observation (e.g. male/female or average/maximum lifespan). This has significantly increased the number of observations originating from Build 16 of the GenAge Database.

AgeFactDB makes an attempt to unify gene symbols. The shortest preferred name suggested by the Gene/Protein Synonym Database—GPSDB (16) is used as (unified) AgeFactDB gene symbol. In the GPSDB, information from other databases such as UniProtKB/SwissProt or Wormbase is collected. Detailed information can be found by following the GPSDB link. In addition to the unified gene symbol, AgeFactDB provides information on synonyms. Therefore, when searching for a gene in AgeFactDB it is not necessary to know the unified gene name in advance, a search will succeed for any of the synonym names including the name used in the source databases.

Data validation is a major issue for all databases. This is especially a problem if the major body of information is contained in free-text descriptions because in this case there is no agreed data structure that could draw attention to inherent inconsistencies and errors. In this context, our data integration approach appears to be very helpful for error identification. For example, one can easily generate a list of observations sorted by the lifespan change given in %, which can then be compared to the qualitative classification (increased, decreased and no statistical significant effect). With this approach, we have already been able to identify a number of inconsistencies. All modifications of ageing factor names and other data are described in the release information.

## DATABASE INTERFACE

AgeFactDB can be accessed either by browsing through predefined lists or by searching.

The available predefined lists include a comprehensive compilation of all ageing factors but there are also separate lists for the three ageing factor types gene, compound and other ageing factors. Moreover, a list of homologues to experimentally known ageing-related genes is provided. In addition to the ageing factor lists also observation lists are available.

Currently, a quick search option is implemented. The search can be performed for one or more search terms where terms can be combined by either a logical AND or OR, with phrase and wildcard searches also possible. Moreover, the search space can be reduced. Examples are the search in ageing factor names including synonyms, PubMed IDs and Medical Subject Headings assigned to references. Finally, the search can also be restricted to find ageing factors or observations from one or more source databases or with specific type(s) of ageing-relevant evidence.

If a valid AgeFactDB ID (ageing factors: AF_nnnnnn; observations: OB_nnnnnn; fixed length, each n represents a digit) is entered as single term, the search is redirected directly to the actual ageing factor or observation page. Compared to the predefined lists the search results lists contain a special column indicating the search term occurrence. Initially, only a limited number of occurrence details are displayed for each ageing factor or observation, but the display can be expanded to show the full details. For the search results, there are two display modes, the ageing factor mode and the observation mode. Finally, the search results are categorized according to species and ageing factor type, as well as to search term occurrence. This allows the quick generation of refined subset lists, such as results for a particular species. An example of the search output is given in Supplementary Figure S1, with the header and one of the ageing factors found by searching for rapamycin. The header displays the refinement options and on the right side, the quick search interface. Note, that this interface does not include all search options. A more detailed description can be found on the Help page. To show only two examples, search term[pubmed] and search term[mesh] perform a search for PubMed IDs and for a search term occurrence in the PubMed Medical Subject Headings only. As mentioned earlier, the search term occurrence is indicated for all observations or ageing factors found. All the predefined and search result lists have a hide/show option for each column, with columns being freely movable throughout the table. With a search term or its negation one can filter the output to display only specific rows. Finally, the list can be sorted by each individual column or a combination of several columns in ascending or descending order.

The observation pages are elementary information units of AgeFactDB. They are colour-coded according to the following rules:

**Table:** 

(1) Light green:	Ageing relevance experimentally confirmed.
(2) Yellow green:	Ageing relevance confirmed but no ageing factor assigned (lifespan data for a population or species without applying interventions).
(3) Red:	Effect of an assumed ageing factor experimentally studied but no significant effect found.
(4) Blue:	Computationally derived homology data.

Ageing factor pages are also colour-coded, but here the situation is more complex. As already mentioned, these pages typically summarize more than one observation and these observations may be of a different type and thus a different colour code. In this case, we adopt the following rules. If at least one observation belonging to the specific ageing factor is green then the colour code for this page is green even though there may be also red or blue observations. In this case we adopt the rule that the most important observation colour is chosen as ageing factor colour. This is always the colour with the lowest number in the order given above.

Observation pages currently contain three parts: General Description, References and Sources. Ageing factor pages currently contain six parts: Description, Protein Information, Observations, Sequences, References and Sources. The Protein Information and the Sequences parts are only available for genes and not for compounds or other ageing factors.

In Supplementary Figures S2A and B, two parts of the ageing factor page for the *C. elegans* gene *aak-2* are shown. Supplementary Figure S2A shows the header and the protein information part. In the header it can be seen that there are 38 observations in the database. Among the 37 experimental studies, there are 11 for which no effect was found and 24 with an either increasing or decreasing lifespan. According to our rules the most important observations related to ageing relevance are the green ones. Therefore, the ageing relevance bar in the upper part of the page is coloured in green. The little boxes on the left bar side indicate that there are also other observations. In Supplementary Figure S2B, the results of homology analysis for this gene are shown. The HomoloGene homology group includes 20 genes and 2 of them are already known from experimental studies as being ageing-relevant.

## OUTLOOK

The first aim of our future work will be the integration of further data from general databases, ageing-related databases or directly from the scientific literature. The second aim is data curation. We have started a validation process for the data from other ageing-related databases within the AgeFactDB environment and were already able to remove a number of inconsistencies and errors. However, this will be an ongoing task. In addition, user interface improvements are planned. We want to expand the export capabilities by providing an XML export option for each individual ageing factor and observation page. Along the same line we will establish a so-called RESTful interface (17). An advanced search option is under development and will extend the available quick search options. We have already implemented the basis for a change report option that is supposed to report all changes between two time points on different levels of detail. This would help, for example, to find out exactly what has changed since the last visit of the database. And besides this we also want to provide a history option that will enable the retrieval of the data for an ageing factor or observation that was available at any earlier time point.

## SUPPLEMENTARY DATA

Supplementary Data are available at NAR Online.

## FUNDING

German Ministry for Education and Research (Bundesministerium für Bildung und Forschung—BMBF) within the GerontoSys initiative [0315581]. Funding for open access charge: Leibniz Institute for Age Research – Fritz Lipmann Institute, Beutenbergstrasse 11, D-07745 Jena, Germany.

*Conflict of interest statement*. None declared.
